# Parallel Computing of Patch-Based Nonlocal Operator and Its Application in Compressed Sensing MRI

**DOI:** 10.1155/2014/257435

**Published:** 2014-05-20

**Authors:** Qiyue Li, Xiaobo Qu, Yunsong Liu, Di Guo, Jing Ye, Zhifang Zhan, Zhong Chen

**Affiliations:** ^1^Departments of Communication Engineering and Electronic Science, Fujian Provincial Key Laboratory of Plasma and Magnetic Resonance Research, Xiamen University, Xiamen 361005, China; ^2^School of Computer and Information Engineering, Xiamen University of Technology, Xiamen 361024, China

## Abstract

Magnetic resonance imaging has been benefited from compressed sensing in improving imaging speed. But the computation time of compressed sensing magnetic resonance imaging (CS-MRI) is relatively long due to its iterative reconstruction process. Recently, a patch-based nonlocal operator (PANO) has been applied in CS-MRI to significantly reduce the reconstruction error by making use of self-similarity in images. But the two major steps in PANO, learning similarities and performing 3D wavelet transform, require extensive computations. In this paper, a parallel architecture based on multicore processors is proposed to accelerate computations of PANO. Simulation results demonstrate that the acceleration factor approaches the number of CPU cores and overall PANO-based CS-MRI reconstruction can be accomplished in several seconds.

## 1. Introduction

The slow imaging speed is one of major concerns in magnetic resonance imaging (MRI). Compressed sensing MRI (CS-MRI) has shown the ability to effectively reduce the imaging time. Numerous researches have been conducted on CS-MRI [1–4] in the past few years. As one key assumption of CS-MRI, MR images are sparse in some transform domains [5, 6] and the sparsity seriously affects the reconstructed images. Unlike using predefined basis, such as wavelets, to represent MRI images, self-similarity of an MR image has been introduced into CS-MRI [7–10] with the help of nonlocal means (NLM) [11] or block matching and 3D filtering [12]. By modeling the similar patches embedded in MR images with a linear representation, a patch-based nonlocal operator (PANO) [13] has been shown to outperform typical total variation, redundant wavelets, and some other methods. PANO can be viewed as an alternative form of the block matching 3D frames [14]. It explores the similarities of nonlocal image patches and allows integrating conventional transforms such as wavelet, discrete cosine wavelet, or discrete Fourier transform to further exploit the sparsity of grouped similar image patches.

However, PANO usually suffers from massive computations because of high overlapping among the patches. Fortunately, learning patch similarities and forward and backward transformations among grouped patches are independent of each other. This allows reducing the reconstruction time by taking the advantage of the technology of parallel computing [15, 16]. In this paper, we design a parallel architecture to accelerate the computations of PANO. There are two main steps in PANO [13], learning similarities and performing 3D wavelet transform on grouped patches. Simulation results demonstrate that the parallel architecture can effectively accelerate the computation speed by making use of multiple CPU cores. This parallel architecture is also widely applicable to the self-similarity-based image reconstruction methods.

## 2. Methods

As mentioned above, there are two major steps in PANO, learning similarities and performing 3D wavelet transform on grouped patches.

**Figure 1:**
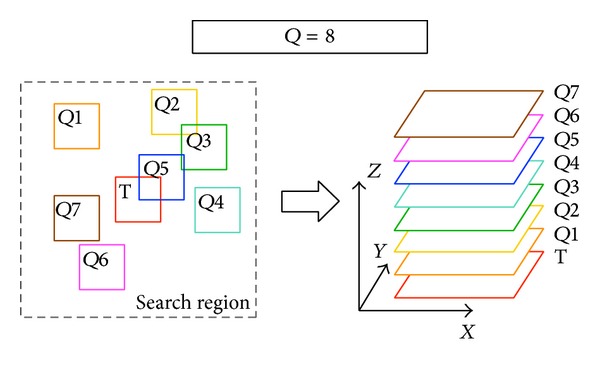
Search* Q-*1 = 7 similar patches in the search region and stack them with the basic patch T into a 3D cube.

We first review the process of learning similarities. For a specified patch, PANO is to find* Q-*1 similar patches (the shorter the Euclidean distance is, the more similar they are) in a search region centered in the specified patch [13]. After the similarity information of a patch is learnt, the* Q* patches (including the specified patch and* Q-*1 similar patches) will be stacked into a 3D cube from the most similar one to the least, as is shown in Figure 1.

As 3D cubes are created, 3D wavelet transform can be applied to them. First, perform 2D forward wavelet transform in X-Y dimension and then do 1D forward wavelet transform in Z dimension. After the forward wavelet transform is done, a soft threshold operator is applied. Following 3D backward wavelet transform, 3D cubes are assembled back to the image.

The two major steps in PANO are both patch-based, and each patch can be handled independently. Therefore, parallel computing can be a good solution to accelerate the computations. More and more image processing applications that exhibit a high degree of parallelism are showing great interests in parallel computing [16]. Parallelization can be easily implemented on multicore central processing unit (CPU) with existing application program interfaces (APIs) such as POSIX threads and OpenMP [15]. Meanwhile, many applications are making use of graphic processing unit (GPU) [17], which is good at doing complex computations. Due to the simplicity, flexible interface, and multiplatform supports, we choose OpenMP as our API to develop parallel computing program on CPU, which is the common configuration for personal computers.

### 2.1. Parallelization on Learning Similarities

**Figure 2:**
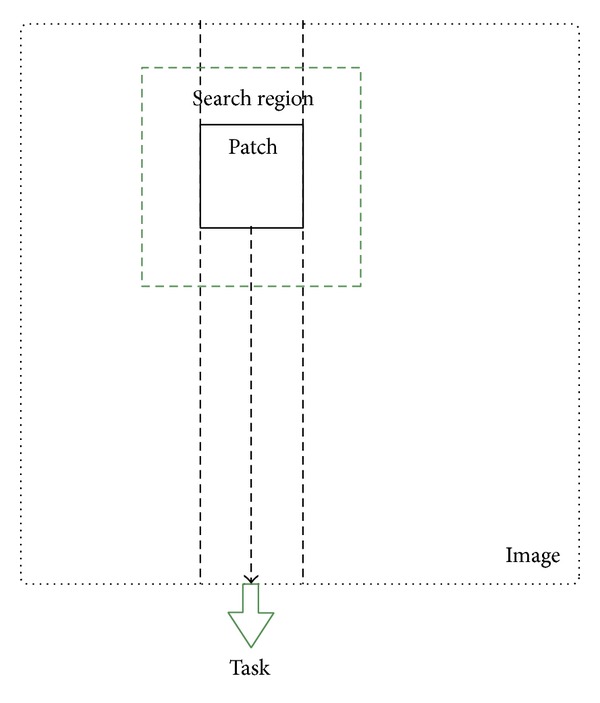
Generating tasks according to image columns. One task corresponds to one column of patches.

In order to make full use of a multicore CPU, tasks should be properly generated and assigned to CPU cores [16]. If there are too many tasks, CPU cores will switch frequently to handle the tasks and the cost for switching between CPU cores is expensive. Therefore, generating a task that is based on a single patch is suboptimal. Instead, generating a task that processes a serial of image patches in one column or one row can be better, as is shown in Figure 2. Because generating tasks according to columns is the same as according to rows, we choose columns as an example in this paper. Computations in one task are serial while each task will be processed in parallel. After one task is done, computations on the patches of the corresponding column will be finished. That is, all the similarity information for the patches in that column is learnt. CPU cores can be effectively utilized when the number of columns in an image is much larger than the number of CPU cores in a personal computer.

### 2.2. Parallelization on 3D Wavelet Transform on Cubes

**Figure 3:**
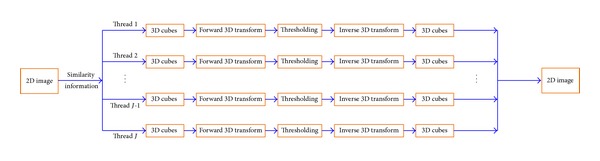
Parallel architecture of 3D wavelet transform on cubes.

After similarity information is learnt, 3D cubes can be easily created, as is shown in Figure 1. The 3D wavelet transform on cubes shares the same parallel architecture as Section 2.1. One task gets out the similarity information of patches in one column. After cubes are created according to the learnt similarity information, wavelet transforms will be applied on them. Computations on cubes within each column of patches will be serial and tasks will be processed in parallel, as is shown in Figure 3.

## 3. Results

Experiments are conducted in three aspects. First, the effectiveness of the parallel architecture on a personal computer is discussed. Second, the performance on a professional workstation with 100 CPU cores will be explored. At last, a complete process in PANO-based CS-MRI will be accomplished to see how much the improvement will be in iterative CS-MRI reconstructions. Every experiment is repeated five times, and the reported computation time is the average of them.

### 3.1. Parallelization Improvement on a Personal Computer

**Figure 4:**
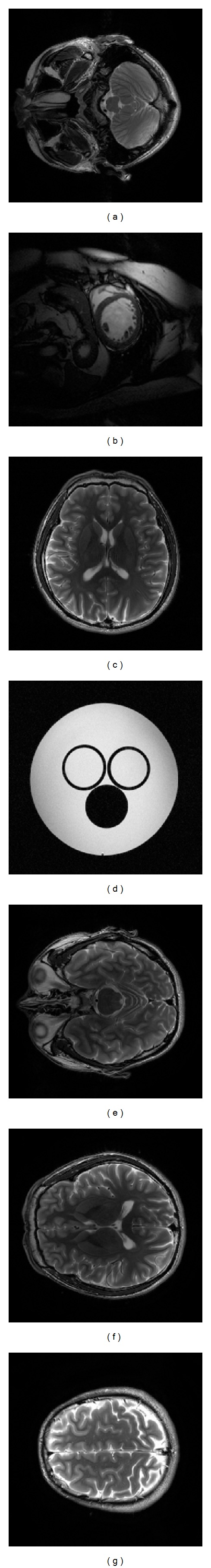
Dataset. (a), (c), (e), (f), and (g) T2 weighted MR brain images, (b) one frame of cardiac MR images, and (d) a water phantom image.

The experiments are conducted on DELL T1700 personal workstation with E3-1225v3 CPU (4 cores, 3.2 GHz). 64-bit Windows 7 is our operating system. MATLAB version is 2013B with MEX compiler from Visual Studio 2012. Patch size is set to be 8 × 8, search region is 39 × 39, sliding step is 1, and the number* Q* is 8. Sliding step is a moving step to select the next patch. Haar wavelet is chosen as the sparsifying transform due to its efficiency and simplicity. Seven images shown in Figure 4 are tested. Figures 4(a), 4(c), 4(e), 4(f), and 4(g) are T2 weighted brain images acquired from a healthy volunteer at a 3T Siemens Trio Tim MRI scanner using the T2 weighted turbo spin echo sequence (TR/TE = 6100/99 ms, 220 × 220 mm field of view, and 3 mm slice thickness).  Figure 4(b) is a cardiac image downloaded from Bio Imaging Signal Processing Lab [18, 19].  Figure 4(d) is a water phantom image acquired at 7T Varian MRI system (Varian, Palo Alto, CA, USA) with the spin echo sequence (TR/TE = 2000/100 ms, 80 × 80 mm field of view, and 2 mm slice thickness).

#### 3.1.1. Accelerating in Learning Similarities

**Figure 5:**
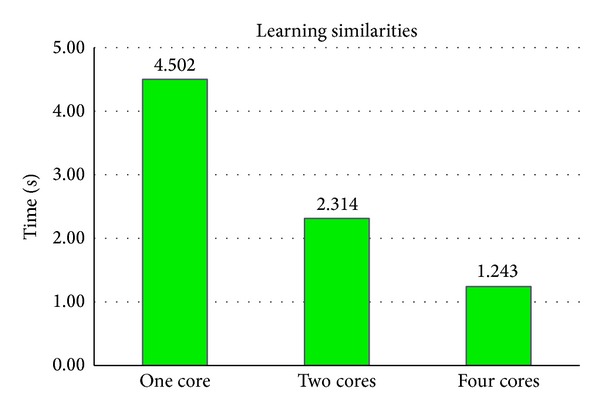
Computation time of learning similarities for Figure 4(a) with different number of CPU cores in use.

As is shown in Figure 5, computation time for learning similarities of Figure 4(a) is effectively decreased with the increasing number of CPU cores in use. It demonstrates that the parallelization is effective. However, the accelerating factor cannot be exactly the same as the number of CPU cores. One reason is that it needs to create and assign tasks to CPU cores, and this activity requires extra time. Furthermore, tasks may not be finished exactly within the same time. Therefore, it is reasonable that the accelerating factor is between 3 and 4 with 4 CPU cores.

#### 3.1.2. Accelerating in 3D Wavelet Transform on Cubes

**Figure 6:**
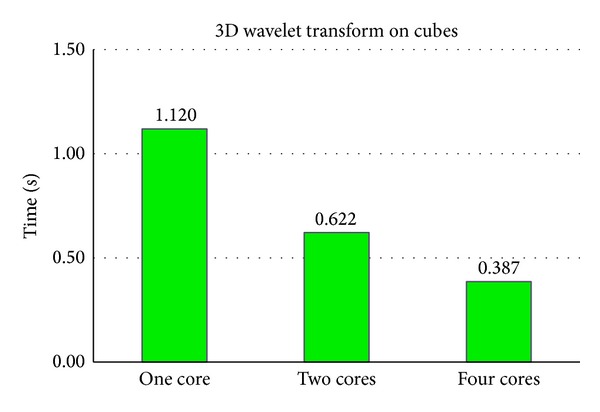
Computation time of 3D wavelet transform on cubes for Figure 4(a) with different number of CPU cores in use.

As is shown in Figure 6, parallelization on 3D wavelet transform on cubes of Figure 4(a) is also very effective. Besides the cost introduced in Section 3.1.1 for parallelization, it also needs time to create cubes and reassemble them back to the image. Therefore the accelerating factor is smaller than four when there are 4 CPU cores in use.

#### 3.1.3. Test Results on More MRI Images

**Figure 7:**
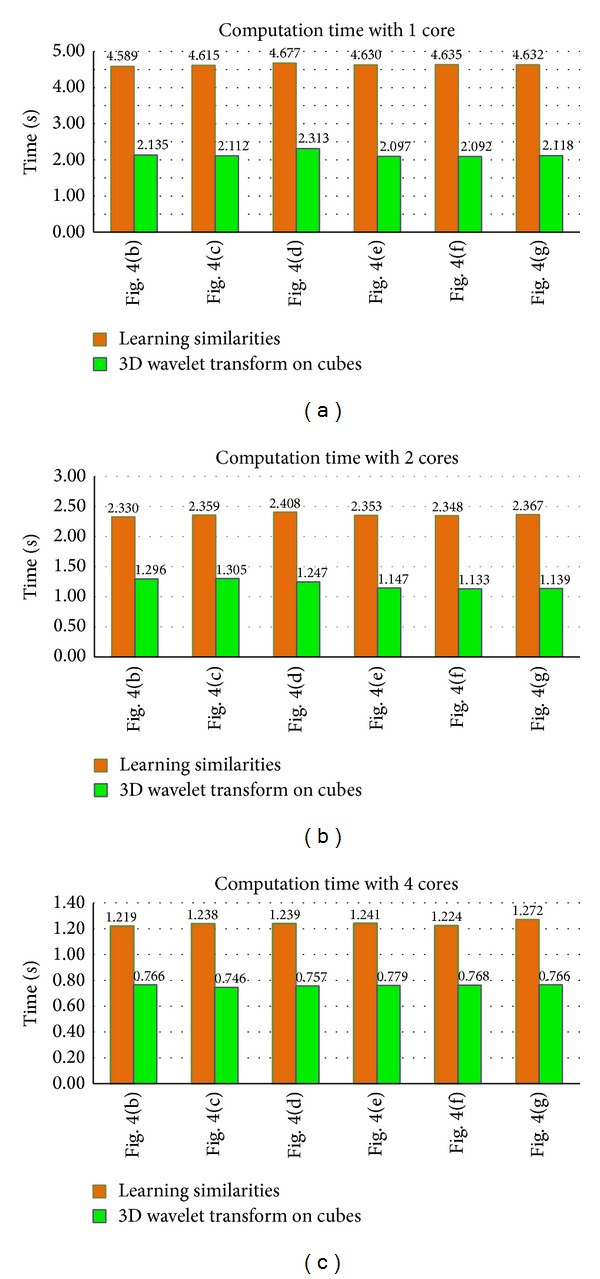
Computation time with various cores for (b), (c), (d), (e), (f), and (g) in Figure 4.

The remaining images in Figure 4 are tested and the computation time is shown in Figure 7. Because Figures 4(b)–4(g) are complex images, the computation time of 3D wavelet transform on cubes is approximately twice the consuming time for Figure 4(a), as real and imaginary parts are computed separately. The computation time is comparable for different images, implying that the computations do not depend on the image structures. Furthermore, as it can be seen in Figures 7(a), 7(b), and 7(c), the acceleration is effective with different images when testing with various CPU cores.

### 3.2. Parallelization Improvement on a Professional Workstation

**Figure 8:**
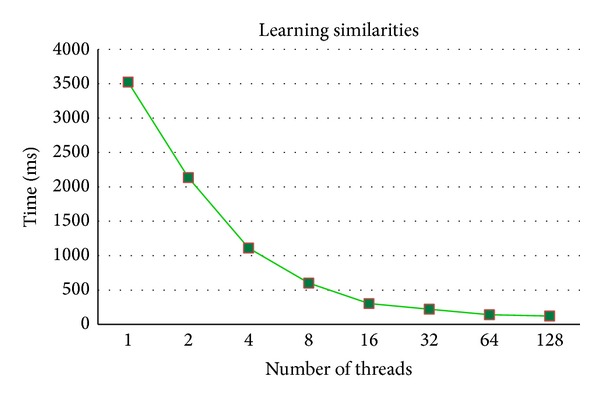
Computation time of learning similarities on a professional workstation for Figure 4(a).

**Figure 9:**
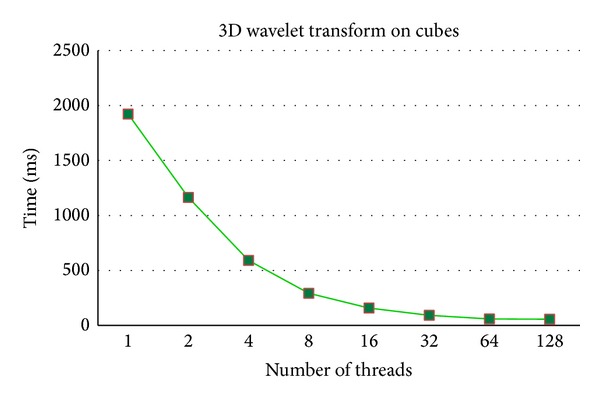
Computation time of 3D wavelet transform on cubes on a professional workstation for Figure 4(a).

The effectiveness on a professional workstation is discussed in this section. Experiments are executed on IBM x3850 with ten E7-8870 CPUs. As shown in Figures 8 and 9, both learning similarities and 3D wavelet transform on cubes are effectively accelerated with 100 cores, and 128 threads are tested due to hyperthreading technology [20].

### 3.3. Parallelization Improvement on PANO-Based CS-MRI

The full reconstruction of PANO-based CS-MRI is discussed in this section. Experiments conducted with the sliding steps are 8 and 4, respectively, and 40% of data are sampled. With a typical setting, similarity is learnt twice, and 3D wavelet transform on cubes will be called about 160 times. As introduced in [13, Algorithm 2], setting the times of updating the guide image as 2 will improve results. Therefore similarity is learnt twice. In [13, Algorithm 1], the assembled image needs to be updated many times to get a sufficient large *β*. Besides, the 3D wavelet transform performed on cubes will be called about 160 times.

As is shown in Table 1, both learning similarities and 3D wavelet transform on cubes are accelerated effectively nearly 4 times with sliding step being 4 and 4 CPU cores in use. When sliding step is 8, a full reconstruction can be accomplished in about 3 seconds. Because computations are very fast and the cost for parallelization is relatively expensive, learning similarities are accelerated less than 3 times.

## 4. Conclusion

In this paper, the patch-based nonlocal operator (PANO) has been parallelized to accelerate its two major steps, learning similarities and performing 3D wavelet transform on grouped similar patches. Experiments conducted on both the personal and professional computers have proven the effectiveness and applicability of the parallel architecture. Results demonstrate that a full PANO-based compressed sensing MRI reconstruction can be accomplished in several seconds. The parallel architecture of PANO is also applicable for other image reconstruction problems [21]. In the future, how to maximize the acceleration for computations of PANO on 3D imaging with multicore CPUs and GPUs will be developed.

## Figures and Tables

**Table 1:** Computation time of PANO-based CS-MRI (unit: seconds).

	Sliding step = 8		Sliding step = 4	
	One core	Four cores	One core	Four cores
Learning similarities	0.077	0.039	0.578	0.153
3D wavelet transform on cubes	7.910	2.438	31.684	8.915
Other computations	0.531	0.546	0.560	0.575
Total reconstruction time	**8.518**	**3.023**	**32.822**	**9.643**
